# Nitrification in acidic and alkaline environments

**DOI:** 10.1042/EBC20220194

**Published:** 2023-08-11

**Authors:** Gaofeng Ni, Pok Man Leung, Anne Daebeler, Jianhua Guo, Shihu Hu, Perran Cook, Graeme W. Nicol, Holger Daims, Chris Greening

**Affiliations:** 1 Department of Microbiology, Biomedicine Discovery Institute, Monash University, Melbourne, Victoria, Australia; 2 Institute of Soil Biology and Biogeochemistry, Biology Centre CAS, Ceske Budejovice, Czechia; 3 Australian Centre for Water and Environmental Biotechnology (Formerly AWMC), The University of Queensland, Brisbane, Queensland, Australia; 4 School of Chemistry, Monash University, Melbourne, Victoria, Australia; 5 Univ Lyon, CNRS, INSA Lyon, Université Claude Bernard Lyon 1, Ecole Centrale de Lyon, Ampère, UMR5005, 69134 Ecully, France; 6 Division of Microbial Ecology, Centre for Microbiology and Environmental Systems Science, University of Vienna, Vienna, Austria; 7 The Comammox Research Platform, University of Vienna, Vienna, Austria; 8 Securing Antarctica’s Environmental Future, Monash University, Melbourne, Victoria, Australia; 9 Centre to Impact AMR, Monash University, Melbourne, Victoria, Australia

**Keywords:** archaea, metabolism, nitrification

## Abstract

Aerobic nitrification is a key process in the global nitrogen cycle mediated by microorganisms. While nitrification has primarily been studied in near-neutral environments, this process occurs at a wide range of pH values, spanning ecosystems from acidic soils to soda lakes. Aerobic nitrification primarily occurs through the activities of ammonia-oxidising bacteria and archaea, nitrite-oxidising bacteria, and complete ammonia-oxidising (comammox) bacteria adapted to these environments. Here, we review the literature and identify knowledge gaps on the metabolic diversity, ecological distribution, and physiological adaptations of nitrifying microorganisms in acidic and alkaline environments. We emphasise that nitrifying microorganisms depend on a suite of physiological adaptations to maintain pH homeostasis, acquire energy and carbon sources, detoxify reactive nitrogen species, and generate a membrane potential at pH extremes. We also recognize the broader implications of their activities primarily in acidic environments, with a focus on agricultural productivity and nitrous oxide emissions, as well as promising applications in treating municipal wastewater.

## Introduction

During aerobic chemolithoautotrophic nitrification, microorganisms convert ammonia (NH_3_) to nitrite (NO_2_^−^) and then nitrate (NO_3_^−^) [1]. The first step is mediated by ammonia-oxidising archaea (AOA) and ammonia-oxidising bacteria (AOB), while the second step is mediated by diverse lineages of nitrite-oxidising bacteria (NOB) [2]. It has also recently been discovered that some *Nitrospira* species, known as comammox bacteria, mediate complete oxidation of ammonia to nitrate [3,4]. Collectively, these microorganisms support key steps in the biogeochemical cycling of nitrogen on the earth and play important ecological roles in diverse terrestrial and aquatic ecosystems, as well as engineered ecosystems such as wastewater and drinking water treatment plants.

**Figure 1:**
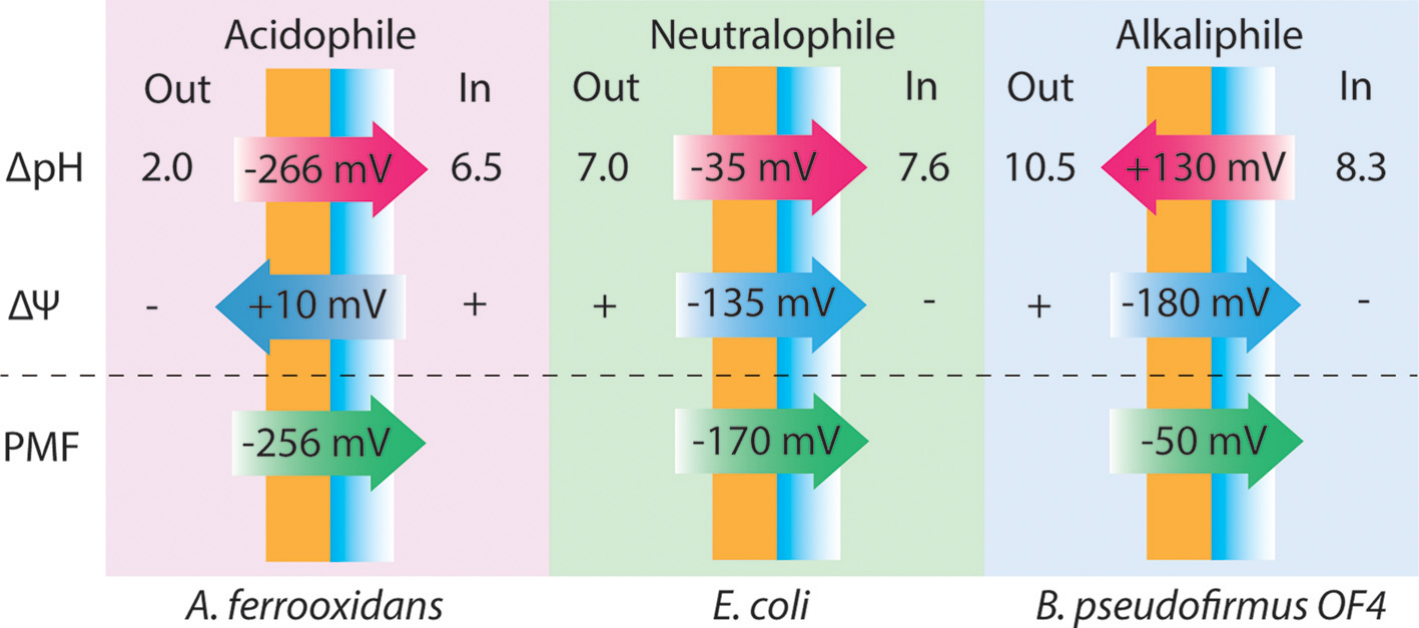
Differences in proton motive force composition under acidic, neutral and alkaline conditions. ΔpH contributes to proton motive force (PMF) generation under acidic and neutral conditions, and detracts from proton motive force under alkaline conditions at 25°C. This is illustrated with examples from the obligate acidophile *Acidithiobacillus ferrooxidans*, the neutralophile *Escherichia coli*, and facultative alkaliphile *Bacillus pseudofirmus* OF4; adapted from Krulwich *et al.*, 2011 [5].

The maintenance of a circumneutral intracellular pH, termed ‘pH homeostasis’, is crucial in any living cell [5–7]. This is because most proteins have distinct ranges of pH within which they can function, while pH also affects the structure of nucleic acid and many other biological molecules [7]. pH also plays a central role in cellular bioenergetics since the establishment of proton-motive force (PMF) depends on both the electrical gradient (membrane potential, ΔΨ) and pH gradient (ΔpH, i.e. pH_in_ − pH_out_), as per the equation PMF = ΔΨ − 59ΔpH at 25°C [5]. It is therefore vital for microorganisms to sense pH in their milieu and maintain intracellular pH homeostasis [5,8]. Most microorganisms have pH optima between 5 and 9, while a small but significant proportion of microorganisms grow optimally at pH values below 5 or greater than 9 [9]. At low pH, PMF is primarily made up of ΔpH, while ΔΨ detracts from PMF formed by ΔpH. At high pH, alkaliphilic and alkalitolerant microorganisms maintain a higher ΔΨ component of the PMF than neutralophiles or acidophiles because the ΔpH component is reversed and detracts from the PMF [5] (Figure 1).

**Figure 2:**
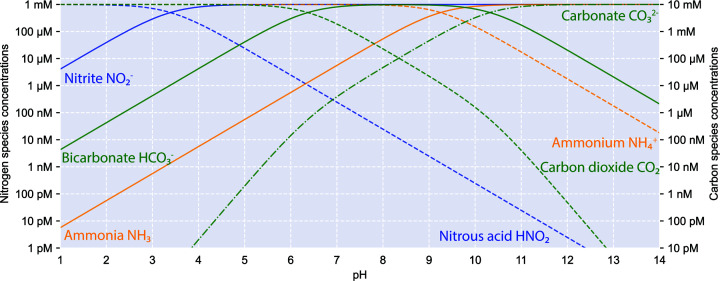
Comparisons of concentrations of nitrogen and carbon sources for nitrifying microorganisms from pH 1 to 14. These comparisons are based on p*K*_a_ values (NH_4_^+^/NH_3_ = 9.25, HNO_2_/NO_2_^−^ = 3.39, CO_2_/HCO_3_^−^ = 6.37, HCO_3_^−^/CO_3_^2−^ = 10.32), calculated using 1 mM total concentration for ammonia (orange, solid) and ammonium (orange, dashed); nitrite (blue, solid) and nitrous acid (blue, dashed); and 10 mM total concentration for bicarbonate (green, solid), carbon dioxide (green, dashed) and carbonate (green, dash dot).

In addition, the shifting equilibrium between acid–base conjugates (determined by acid dissociation constant p*K*_a_) of growth substrates at pH extremes imposes nutritional and toxicity stresses on nitrifying microorganisms. Ammonia is the presumed substrate for ammonia monooxygenase [10–14] and it is more abundant than ammonium at pH conditions < 9.25 (p*K*_a_ NH_4_^+^/NH_3_ = 9.25, at 25°C and zero ionic strength, same conditions used herein). Similarly, nitrous acid (HNO_2_) dominates over nitrite (NO_2_^−^) below pH 3.39 (p*K*_a_ HNO_2_/NO_2_^−^ = 3.39), while bicarbonate (HCO_3_^−^) dominates over carbon dioxide (CO_2_) above pH 6.37 (p*K*_a_ CO_2_/HCO_3_^−^ = 6.37) (Figure 2) [15]. pH drastically influences the speciation of these compounds, as decreasing one pH unit would result in 10 times higher acid/base ratio for each conjugating pair. Thus, the effect of pH on nitrifying microorganisms includes the availability of energy and carbon sources, as well as the toxicity of reactive nitrogen species such as nitrous acid.

While the process of nitrification has been studied extensively owing to its biogeochemical and industrial significance, most of our understandings are centered towards neutralophilic nitrifiers or near-neutral environments. An increasing number of culture-dependent and culture-independent studies (summarised in Table 1) have demonstrated that nitrification is active at pH extremes and identified the microorganisms mediating these processes. However, our insights on how nitrifying microorganisms have adapted to acidic and alkaline pH remain rudimentary. This topic has not previously been reviewed, with the exceptions of a consideration of the pH dependency of enzymatic processes regulating N_2_O production in wastewater treatment systems [16], one study in 2012 that reviewed progress in mechanistic understanding that could explain the dominance of AOA over AOB in certain acidic soils [17], and prior reviews focusing primarily on the distribution and diversity of nitrifiers in acidic soils [18–20]. In the following sections, we describe the process of nitrification, as well as the pH preferences and cellular adaptations of nitrifiers, in diverse acidic and alkaline ecosystems. For a description of the distribution of nitrifiers in acidic and alkaline habitats, we refer to Table 1.

**Table 1:** Overview of reported nitrification in acidic (≤5.5) and alkaline (≥8.5) environments, including information on the identified nitrifier groups and organisms

pH range*	Environments	Nitrifier group	Representative taxa	Technique/type of study**	Citation
2.0–7.0	Acidic tea field	AOB	*Ca*. Nitrosoglobus terrae	F, I, B, Q, A, O, K, T	[21]
2.2–5.4	Laboratory reactor	AOB	*Nitrosococcus*-like	I, B, A, T	[22]
2.5–7.0	Laboratory reactor	AOB	*Ca*. Nitrosacidococcus tergens	I, B, A, O, M, K	[23]
2.5–7.0	Laboratory reactor	AOB	*Ca*. Nitrosacidococcus urinae	I, B, A, K	[24]
2.9–7.0	Agricultural soil	AOB	*Nitrosococcus*-like	F, I, B	[25]
3.0–10.0	Laboratory incubation	AOB, AOA	*Nitrosomonas europaea, Nitrosomonas multiformis, Nitrosospira briensis*, and *Nitrosopumilus maritimus*	I, H	[26]^†^
3.2–6.6	Agricultural soil	AOB	*Nitrosomonas*	F, B, Q	[27]
3.3–4.5	Permafrost environment	AOA, AOB	NA	F, B, Q, S, O, T	[28]
3.6–4.5	Heathland soil	AOB, NOB	*Nitrosospira* spp. AHB1, *Nitrobacter* spp. NHB1	F, I, B, M	[29–31,32,33]
3.7–8.8	Soil	AOA	NA	F, A, T	[34]
3.8–4.3	Forest soil	AOA	NA	F, B, A, Q	[35]
3.8–5.3	Agricultural and forest soil	AOA	*Nitrosotalea*-like	F, I, B, D, O	[36]
3.8–5.7	Laboratory reactor	AOB	*Ca*. Nitrosoglobus	I, B, A, T	[37]
3.8–6.2	Agricultural soil	AOA, AOB	NA	F, I, A, Q, T	[38]
3.8–8.0	Soil	AOA	NA	R	[17]
<4.0–6.0	Agricultural soil	AOB, NOB, comammox	*Nitrosospira*, canonical and comammox *Nitrospira*	F, B, I, A, M	[39]
4.0–4.1	Forest soil	AOA	NA	I, B, A, Q, T	[40]
4.0–4.3	Agricultural and restored soil	AOA, AOB	*Nitrosomonas*, *Nitrosospira*	F, B, A, Q, S	[41]
4.0–5.5	Agricultural soil	AOA	*Ca*. Nitrosotalea devaniterrae	F, B, I, A, M, O, T	[42–45]
4.0–7.5	Laboratory cultivation	AOB	*Nitrosospira* sp. NPAV	I, B, Q	[46]
4.0–5.8	Forest soil	AOA, AOB, NOB	*Ca*. Nitrosotalea, *Nitrosospira, Nitrospira*, *Nitrobacter*, *Nitrosococcus*, *Nitrosospira*	F, I, B, A, O, T	[47]
4.1–4.9	Forest, grassland, and wetland	AOA, NOB	NA	F, O	[48]
4.1–5.2	Forest soil	AOA, AOB	NA	F, B, A, Q, T	[49]
4.2–4.5	Agricultural soil	AOA, AOB	NA	F, B, A, Q, T, D	[50]
4.2–7.5	Glacier foreland soil	AOA	NA	F, A, Q	[51]
4.3	Laboratory reactor	AOB	*Ca*. Nitrosoglobus	I, B, A, Q	[52]
4.3–5.2	Forest soil	NOB	*Nitrobacter* spp. IOacid	F, I, B, M	[53]
4.7–5.6	Volcanic soil	AOA, AOB	*Nitrososphaera*, *Ca*. Nitrosotalea, *Nitrosospira*, *Nitrosomonas*	F, B, A, T	[54]
4.8–6.4	Peat land	AOA, AOB, NOB	NA	F, A, T	[55]
4.8–6.9	Agricultural soil	NOB	*Nitrospira*, *Nitrobacter*	F, B, A, Q, O, T	[56]
5.0	Laboratory reactor	AOB	*Ca*. Nitrosoglobus	I, B, A, K	[57]
5.0–7.0	Laboratory reactor	AOB	*Ca*. Nitrosoglobus	I, B, A, K	[58]
5.0–8.0	Laboratory reactor	AOB	*Nitrosomonas europaea*	I, B	[13,59]
5.0–9.5	Soil	AOA	NA	F, A, Q	[60]
5.2–5.9	Agricultural soil	AOA, AOB	NA	F, I, Q	[61]
5.5–8.5	Laboratory cultivation	AOA, NOB	*Nitrosocosmicus oleophilus*, *Nitrosotenuis chungbukensis*, *Nitrosomonas europaea, Nitrobacter winogradskyi*	I, S, B, A, T	[62]
5.5–8.0	Hot spring	NOB	Chloroflexota	F, I, Q, A, O, M	[63]
8.2–8.8	Glacier soil	NA	NA	F, B, A, T	[64]
8.9	Laboratory reactor	NA	NA	I, B, M	[65]
9.5–10.2	Soda lake	NOB	*Nitrobacter alkalicus*	F, I, B, A, M	[66]
9.7	Saline-alkaline lake	NA	NA	F, B, S	[67]
9.8	Soda lake	AOA, AOB	*Nitrosomonas*	F, B, A, M	[68]
9.0–11.0	Soda lake	AOB, NOB	*Nitrosomonas*, *Nitrobacter*	R	[69]
8.9–10.5	Saline-alkaline lake	NOB	canonical *Nitrospira, Ca*. Nitrospira alkalitolerans	F, I, B, A, O, M	[70]
9.6–10.4	Soda lake	AOA	NA	F, B, A	[71]
9.5–11	Soda lake	AOB, NOB	*Nitrosomonas*, *Nitrobacter*	R	[72]
7.6–11.0	Saline-alkaline lake	AOA, NOB, and comammox	*Nitrosocosmicus, Nitrososphaera*, canonical and comammox *Nitrospira*	F, B, A, S	[73]
∼11.3	Soda lake	AOB, NOB	*Nitrosomonas halophilus*, *Nitrobacter alkalicus*	R	[74]

* Sorted based on lowest or highest reported pH for the detection of nitrification or nitrifiers in acidic or alkaline environments, respectively

**Abbreviations of techniques or study type: F: Field campaigns I: Isolation or cultivation B: Biochemical assays and physiological studies Q: Auantitative polymerase chain reaction (qPCR) A: Amplicon-based sequencing analysis using genes such as 16S rRNA gene, *amoAB* and nitrite oxidoreductase (*nxrAB*) O: Omics and meta-omics including genomics, transcriptomics, and proteomics M: Microscopy including electron microscopy and fluorescence *in situ* hybridization (FISH) S: Stable isotopic tracing T: Statistical analysis K: Kinetics D: DNA stable-isotope probing (DNA-SIP) H: Thermodynamic investigation R: Review article

† This study applies calorimetric and potentiometric titrations to thermodynamically characterise cell surfaces of AOB and AOB from pH 3 to 10, which is beyond the growth pH range of subject microorganisms.

## Ecological distribution

To date, there are over 50 studies reporting nitrification activities from a range of acidic and alkaline environments (Table 1). At acidic pH, nitrifiers have been reported to occur in agricultural, forest, coastal, permafrost, and volcanic soils, as well as in various reactor systems treating municipal or mining wastewaters. Nitrification has been less frequently investigated at high pH, but it is known to occur in certain alkaline saltmarshes, saline-alkaline and soda lakes, and bioreactors. The habitats with reported nitrification activities at the lowest pH (2.0) include acidic tea fields and laboratory bioreactors [21–23], while the activity at the highest pH (11.3) detected was in soda lakes [74].

### Acidic ecosystems

Some of the highest rates of soil nitrification are found in acidic soils (pH < 5.5) that constitute approximately 30% of the world’s ice-free land [75]. The majority of these soils are naturally acidic, and approximately 5% are used for arable crops [75]. AOA are generally the predominant nitrifiers in such soils, which makes them a crucial subject for investigation [19,75,34]. The best-studied acidophilic AOA belong to the genus *Ca*. Nitrosotalea [42], which is currently represented by three cultivated strains (*Ca*. Nitrosotalea devaniterrae, *Ca*. Nitrosotalea sinensis and *Ca*. Nitrosotalea okcheonensis) with growth pH restricted to 4 and 5.5 (optima between 4 and 5) [42–45]. This is supported by a regional and global phylogenetic analysis of archaeal *amoA* gene distribution [34,76], which demonstrate that most lineages of *Ca*. Nitrosotalea are distributed in acidic environments. In addition, these studies revealed that clades of the *Nitrososphaerales* (γ and ζ [76]) are also consistently found in acidic soils, revealing patterns of niche speciation driven by low pH in AOA. This view is supported by a study using Bayesian comparative phylogenetics to investigate co-evolutionary relationships between *amoA* and soil characteristics, which shows that pH is a strong driver for AOA speciation [77]. However, no higher-level AOA clades that are specific to neutral or near-alkaline pH have been identified so far [76].

AOB can outnumber AOA in certain acidic soils and man-made ecosystems containing high levels of ammonium or urea [78,79] (Table 1). Cultivated neutralophilic AOB in the order of *Burkholderiales* within the *Gammaproteobacteria* (formerly placed within *Betaproteobacteria*) have been demonstrated to grow in acidic conditions when using urea as a substrate [29, 30, 46] or when growing in aggregates [31] or biofilms [59]. To date, the most acid-tolerant ammonia oxidizers characterised belong to the two gammaproteobacterial AOB isolates, *Ca*. Nitrosoglobus terrae TAO100 [21] and *Ca*. Nitrosacidococcus tergens RJ19 [23]. Both are most closely related to the neutralophilic marine AOB genus *Nitrosococcus* [23,21]. While both AOB have an optimum growth pH ∼6, they can mediate ammonia oxidation at pH 2–2.5, with *Ca*. Nitrosacidococcus tergens RJ19 even growing at pH 2.5. Additionally, 16S rRNA gene-based surveys in bioreactors treating wastewater suggest a much higher diversity of acid-tolerant AOB than currently enriched [22,52,24]. Finally, both AOA and AOB are found to be pioneer microorganisms in acidic volcanic soils [54], with their capacity to autotrophically grow on inorganic nitrogen compounds facilitating primary succession of barren environments [80].

A range of NOB have been identified through culture-independent surveys of various acidic systems such as soil and aquatic ecosystems, geothermal sites, and engineered environments. NOB activities and adaptations have been minimally studied under low pH conditions through bioassays and culture-dependent methods. To date, two acidophilic *Nitrobacter* strains have been isolated: *Nitrobacter* NHB1 from acidic heath soil [30] with an activity range of pH 5.0–7.5 and *Nitrobacter* strain IOacid from an acidic forest soil with maximal nitrite oxidation activity at pH 5.5 (range 4.1–7.2) [53]. Furthermore, canonical (only nitrite-oxidizing) and a comammox clade A *Nitrospira* strain have been enriched from acidic agricultural soils with activity from pH 4 to 6 [81]. To our knowledge, NOB and comammox organisms from acidic environments have not been subject to detailed physiological studies or genomic characterisation.

### Alkaline ecosystems

Nitrification also occurs in saline-alkaline lakes and soda lakes ecosystems, as confirmed by *ex situ* incubations of lake sediments [82,73]. Multiple studies have shown that ammonia oxidisers mediate ammonia oxidation and primary production at high pH in these environments [74,67, 68, 71–73, 83]. *Nitrosomonas halophila* Ans5, the most alkaliphilic AOB known, was isolated from a Mongolian soda lake in 2001 [84]. This bacterium has an optimal pH range of 8.5–9.5 for growth and can maintain growth up to pH 11.3. In 2008, AOB affiliated with *Nitrosomonas* and AOA from *Nitrososphaerales* were suggested to be significant contributors of nitrification in the saline and alkaline Mono Lake, California [68]. Moreover, a recent study implicates both comammox and canonical *Nitrospira*, as well as several AOA phylotypes, in carrying out rapid nitrification in saline-alkaline lakes at pH up to 11 [73]. Analysis of an enrichment culture of the canonical nitrite oxidiser *Ca*. Nitrospira alkalitolerans showed it thrives at pH between 8.9 and 10.3, and is tolerant to hyposaline and subsaline conditions [70]. Five strains from *Nitrobacter* (AN1–AN5) have been isolated from sediments of soda lakes and soda soil at pH 10 with nitrite as the sole electron source; they formed a distinct species cluster denoted *Nitrobacter alkalicus* [84]. Several excellent reviews have highlighted the central role of nitrifying microorganisms in regulating elemental cycling and enabling primary production in alkaline and saline soda lakes [74,72,69]. However, compared to acidic ecosystems, research on nitrification in alkaline ecosystems is scarce. Given the apparent presence and activity of nitrifiers in alkaline environments, future research on the distribution, physiological speciation and adaptation to elevated pH of these nitrifiers is needed to better understand alkaline nitrification and explore its potential applications.

## Physiological adaptations

### Ammonia acquisition and oxidation

**Figure 3:**
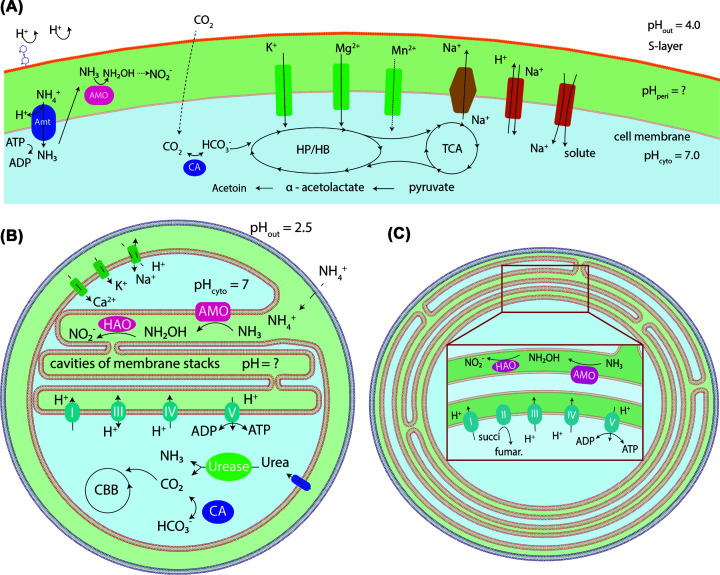
Conceptual diagrams of substrate acquisition and pH homeostasis systems encoded in the genomes of nitrifiers. (**A**) AOA *Ca*. Nitrosotalea devaniterrae based on illustrations in Lehtovira-Morley *et al.*, 2016 [44]. (**B**) The intracytoplasmic membrane stacks based on illustration and electron micrographs of *Ca*. Nitrosacidococcus tergens RJ19 in Picone *et al.*, 2020 [23]. The cation transporters and Na^+^/H^+^ antiporters provide pH homeostasis capabilities. (**C**) Proposed allocations of AMO and HAO on the intracytoplasmic membrane stacks according to electron microgram of *Nitrosomonas halophila* Ans1 [84]. The presence of respiratory complexes is inferred from the model pathway of *N. halophila* Nm1 at Biocyc.org [88]. Respiratory complexes I to V are indicated by roman numerals in panels (B and C); HP/HB, the hydroxypropionate/hydroxybutyrate cycle; TCA, the tricarboxylic acid cycle; CBB, Calvin–Benson–Bassham cycle; CA, carbonic anhydrase.

The active sites of ammonia monooxygenase (AMO) and hydroxylamine dehydrogenase (HAO) are thought to be periplasmic in AOA and AOB (Figure 3A–C) [85]. Our current understanding of comammox *Nitrospira* also favours a periplasmic orientation of AMO and HAO [3,86]. Therefore, the functionalities of these enzymes are likely influenced by environmental pH, as periplasmic pH is likely closer to external pH [5,87]. At acidic pH, ammonia (generally considered to be the substrate of AMO) becomes substantially scarce, posing a major substrate limitation for ammonia oxidisers (Figure 2). Early observations that the AOB *Nitrosospira sp*. AHB1 and *Nitrosospira. sp*. NPAV were able to grow on urea alone, but not on ammonium, at pH < 5.5 have led to the hypothesis that intracellular ureolysis enables ammonia acquisition and oxidation to occur independently of extracellular pH [46,29]. However, *Ca*. Nitrosotalea devaniterrae is not ureolytic [44], and the ability of the AOB *Ca*. Nitrosacidococcus tergens RJ19 and *Ca*. Nitrosoglobus terrae TAO100 to grow at low pH in ammonium-fed bioreactors without added urea suggests that ureolysis can play an important, but not essential role, in adaptation to low pH for some ammonia oxidizers. It should be noted that AMO and HAO of known comammox *Nitrospira* are phylogenetically distinct from those of canonical AOB [3,4,86] and their orientation has yet to be proven. This knowledge is necessary to fully understand the impact of pH on bioenergetics in comammox *Nitrospira*.

The obligately acidophilic AOA *Ca*. Nitrosotalea devaniterrae is hypothesized to overcome ammonia limitation at picomolar ranges by actively importing NH_4_^+^ into the cytoplasm [44]. This is accompanied by deprotonation to NH_3_ and diffusion of NH_3_ through the cytoplasmic membrane back into the pseudo-periplasmic space to generate sufficiently high levels of ammonia locally [44] (Figure 3A). Acid-tolerant/acidophilic AOA likely have a competitive advantage in acidic soils, just as they do in the oligotrophic ocean, due to a high ammonia affinity and efficient CO_2_ fixation pathways [14,89,90]. Indeed, *Ca*. Nitrosotalea devaniterrae possesses the highest specific affinity for NH_3_ of all kinetically characterized AOA [14]. Based on simulations, Li and co-authors further proposed that the negatively charged S-layer proteins of AOA may serve as a reservoir for the positively charged NH_4_^+^, thus indirectly increasing the NH_3_ concentration in the pseudo-periplasmic space and around AMO [91,26]. No AmtB-type ammonia transporter could be identified in the genomes of the two AOB species with the highest tolerance to low pH conditions and it has been speculated that these organisms rely on permeases or passive ammonia diffusion across the membrane (Figure 3B) [23].

Unlike AOA, ammonia oxidation in AOB likely occurs primarily around the extensive specialized intracytoplasmic membrane stacks predicted to house enzymes involved in nitrification and respiration to facilitate energy generation (Figure 3B) [23]. The membrane stacks found in many AOB may increase the membrane surface and periplasm volume and provide more space for the ammonia oxidizing enzymes. This would increase the maximal reaction rate at the cellular level and thus the rate of energy generation from NH_3_. This may help acidophilic AOB to survive under strongly NH_3_-limited conditions and provide a competitive advantage over other AOB. It is worth noting that the intracellular localization of intracytoplasmic membrane stacks displays a considerable distinction across different phylogenetic lineages of AOB [92]. For instance, in the AOB *Nitrosomonas halophila*, intracytoplasmic membrane stacks are localized in the immediate periphery of the outer cytoplasmic membrane [84] whereas those in *Ca*. Nitrosacidococcus tergens RJ19 and *Ca*. Nitrosoglobus terrae TAO100 are centered towards the cellular center (Figure 3C) [23,21]. Whether there is link between the cellular allocation of intracytoplasmic membrane stacks and adaptation towards acidic conditions remains to be determined.

### Nitrite acquisition and oxidation

A low pH also presents challenges for nitrite acquisition in NOB. The chemical equilibrium dictates the dominance of HNO_2_ over NO_2_^−^ under acidic conditions (Figure 2), further limiting the availability of nitrite, a transient substrate especially at reduced pH [93,94]. In addition, a high level of HNO_2_ imposes toxicity, nitrosative stress, and structural damage to the cell [95–98]. Both *Nitrobacter* and *Nitrospira* species have been detected in and/or isolated from acidic soils [53,39], where they likely inhabit different niches given their differences in affinity (to substrate NO_2_^−^) [99] and sensitivity (towards the toxic NH_3_ and HNO_2_) [100]. Moreover, micro-niches in soil might provide different pH micro-environments, allowing coexistence of different NOB. Therefore, kinetic data for the mostly uncultured NOB in soils, and physiological and comparative genomic studies of their mechanisms to combat nitrosative stress, are crucially needed to decipher the respective adaptive traits of different NOB lineages in acidic soils and other habitats. At high pH, the NO_2_^−^-HNO_2_ equilibrium is unlikely to be a limiting factor, because NO_2_^−^ is the predominant form over HNO_2_ and therefore HNO_2_ toxicity plays no significant role at high pH (Figure 2). It is worth noting that the capacity of NOB including *Nitrobacter* and *Nitrospira* to use alternative energy sources, including and hydrogen, formate, and acetate, may also enable them to adapt to fluctuations in nitrite availability and fulfill their energetic requirements for intracellular pH homeostasis [101–106]. Indeed, the genome of the NOB *Ca*. Nitrospira alkalitolerans encodes a high-affinity group 2a [NiFe]-hydrogenase to potentially consume atmospheric H_2_, suggesting physiological flexibility as demonstrated in the neutralophile *Nitrospira moscoviensis* [101,105,107].

### Intracellular pH homeostasis

Nitrifying microorganisms adapted to acidic environments use several mechanisms to overcome intracellular acidification resulting from inward proton flow due to the high ΔpH. In a recent study, Wang and colleagues demonstrated that AOA adapted to acidic conditions had horizontally acquired V-type ATPases that extrude cytosolic protons; consistently, heterologous expression of this enzyme in *Escherichia coli* enhanced acid tolerance [36]. Ion transporters and reduced membrane permeability have also been proposed to facilitate pH homeostasis in *Ca*. Nitrosotalea devaniterrae, including a range of cation transporters predicted for K^+^, Na^+^ and divalent cation uptake, a H^+^/Na^+^ antiporter, a carbonic anhydrase that may facilitate pH homeostasis in addition to contributing to carbon fixation, and an α-acetolactate decarboxylase suggested to mediate proton scavenging [44]. This archaeon may possess lipid membranes that are less permeable compared with neutralophilic AOA in addition to increased sugar units on the outside of the S-layer that are suggested to prohibit proton entry (Figure 3A) [44]. Several studies have inferred mechanisms for acidic adaptations of AOB based on genomic analysis, though without further validation [23,21]. The AOB *Ca*. Nitrosacidococcus tergens is proposed to use multiple enzymes for this purpose: carbonic anhydrases to scavenge protons, cation transporters to maintain an inside-positive ΔΨ, and several Na^+^/H^+^ transporters that may contribute to pH homeostasis (Figure 3B) [23]. The AOB *Ca*. Nitrosoglobus terrae TAO100 also encodes cation transporters which may be involved in the creation of an inside positive membrane potential to resist proton flux [21].

At high pH, the energetic challenge is much more severe as ΔpH detracts from PMF and it is therefore crucial to maintain a high ΔΨ with tightly regulated pH and ion homeostasis [5]. Instead of relying solely on PMF for ATP synthesis, the NOB *Ca*. Nitrospira alkalitolerans can potentially conserve energy through a sodium-motive force (SMF) as inferred by the presence of an N-type ATPase and a sodium-dependent NADH:quinone oxidoreductase in the genome next to the canonical complexes of the electron transport chain, in conjunction with the several H^+^/Na^+^ antiporters such as Mrp- and Nha-type antiporters to facilitate pH homeostasis [70].

While complex mechanisms are adopted by different groups of nitrifying microorganisms to regulate intracellular pH at a single cell level, microbes do not live in isolation in nature. Biofilms and aggregates formed by nitrifying communities provide microsites and buffers that may enable nitrification to continue at pH beyond tolerable ranges at the individual level. For example, de Boer et al. reported that aggregated but not individual cells from nitrifying communities in two Dutch acidic heath soils were able to nitrify at pH 4 [31], while Allison and Prosser also observed that biofilm populations of *Nitrosomonas europaea* oxidized ammonia at lower pH than planktonic populations [59]. Consistently, aggregate cell formation of *Ca*. Nitrosoglobus terrae TAO100 is induced under strongly acidic conditions [21] and *Ca*. Nitrospira alkalitolerans cells enriched under high pH densely clustered into consortia [70].

### Nitrifier carbon fixation

As chemolithoautotrophs, nitrifying microorganisms build biomass through energy-demanding CO_2_ fixation pathways. AOA employ a variant of the hydroxypropionate/hydroxybutyrate (HP/HB) cycle. Being the most energy-efficient known aerobic carbon fixation pathway, it facilitates their adaptation to oligotrophic environments [108]. However, the carboxylases involved in this pathway use bicarbonate as a substrate [108], which is limited relative to dissolved CO_2_ at low pH (Figure 1). Some acidophilic AOA may enhance the rates and efficiency of carbon fixation through carbonic anhydrase to enrich intracellular bicarbonate through the conversion of CO_2_; consistently, carbonic anhydrases have been identified in two of four *Ca*. Nitrosotalea genomes [45]. At high pH, importing bicarbonate rather than CO_2_ is more advantageous due to the higher concentration of bicarbonate (Figure 2).

AOB and Chloroflexota NOB adopt the Calvin–Benson–Bassham (CBB) cycle, which uses CO_2_ as the substrate [63]. Despite the higher availability of dissolved CO_2_ at low pH, these nitrifying microorganisms have evolved mechanisms to further facilitate carbon fixation, possibly in part due to the low catalytic and energetic efficiency of the CBB cycle [109]. For instance, the genome of the acid-tolerant AOB *Ca*. Nitrosacidococcus tergens encodes two types of carbonic anhydrases that may support a ‘buffering’ mechanism by forming bicarbonate from CO_2_ that has entered the cytoplasm (with proton consumption) and subsequently converting bicarbonate back to CO_2_ as required by carbon fixation. *Ca*. Nitrosacidococcus tergens and *Ca*. Nitrosoglobus terrae TAO100 contain a urease that breaks down urea to generate CO_2_, in addition to providing NH_3_ as a source of energy [23,21]. On the other hand, the alkaliphilic *Nitrosomonas halophila* contains carboxysome-like organelles [84], which may be responsible for concentrating CO_2_ as suggested for *Nitrosomonas eutropha* C91 [110,111]. Finally, NOB in the genus *Nitrospira* employ the reductive tricarboxylic acid cycle for carbon fixation, which utilizes CO_2_ as the substrate [86,112,113]. The NOB *Ca*. Nitrospira alkalitolerans is likely able to use the sodium gradient between its saline-alkaline environment and the cytoplasm for bicarbonate uptake with a BicA symporter. Its cytoplasmic carbonic anhydrase may then convert the imported bicarbonate to CO_2_ in the cytoplasm for carbon fixation [70]. However, genes encoding these enzymes are also present in the genome of the neutralophilic marine species *Nitrospira marina* [114].

## Applications and implications

### Agricultural land

Agricultural soils accounted for 38.8% of global N_2_O emission between 2007 and 2016 [115]; the proportion and the gross emission are expected to intensify continually in the coming decade driven by increased fertilizer application and population growth [116]. Understanding the link between soil pH, productivity and microbial nitrogen cycling in soil is crucial to mitigating N_2_O emissions and enhancing productivity from agricultural land. Firstly, soil pH and crop productivity are intrinsically linked to nitrifier activities. This is because nitrifiers in agricultural soils compete for ammonium with crops, contribute to soil acidification due to the conversion of ammonia to nitrite in the first step of nitrification, and accelerate nitrogen losses through leaching of the highly soluble end-product nitrate [18]. Secondly, pH is strongly correlated with the emission of N_2_O from soil [117]. This is because both nitrifiers and denitrifiers are drivers of N_2_O emission [118,119], and their activities are strongly influenced by soil pH [120–123]. Soil acidification affects N_2_O emission in opposite ways between nitrifiers and denitrifiers. For instance, low pH impairs the maturation of N_2_O reductase in the denitrifier *Paracoccus denitrificans* [124], therefore, N_2_O emission by denitrifiers is increased when soil pH decreases [125,126], as N_2_O reductase is the only known enzyme able to reduce N_2_O to N_2_ [117]. In contrast, N_2_O emission by nitrifiers decreases as pH decreases because of niche differentiation; low pH in general favours the dominance of AOA over AOB and AOA produce less N_2_O [117,118,127]. N_2_O yields from ammonia oxidation by AOB species (0.1–8%) are generally at least one order of magnitude higher than from AOA species (0.04–0.3%) and comammox bacteria (0.07%) [119], with AOB activity generating approximately double the yield of AOA in soil [127]. Therefore, soils dominated by AOA and comammox *Nitrospira* are predicted to have lower N_2_O emission potentials than soils dominated by AOB activity, which are also associated with high inputs of inorganic ammonium fertilizer [118,128].

Various strategies to inhibit nitrification have been developed, which can be used in acidic agricultural soils to reduce N_2_O emissions and nitrogen loss. The most widely adopted method is the field application of nitrification inhibitors such as dicyandiamide (DCD), nitrapyrin (NP), and dimethylpyrazole phosphate (DMPP). These commercial inhibitors were tested against neutralophilic AOB and developed prior to the discoveries of AOA and comammox, and prior to the isolation of acid-tolerant ammonia oxidisers [19]. Indeed, the efficacy of these inhibitors varies across different groups of nitrifying microorganisms and soils with different pH and physicochemical conditions as reviewed by Li et al. [19] and Beeckman et al. [129]. AOA rather than AOB are often major ammonia oxidisers in agricultural soils [18,130,131] and the improved identification of resident nitrifiers is facilitating the development of targeted approaches to suppress their nitrification activities at various soil pH regimes. For example, a novel potential nitrification inhibitor, quinone imine, has recently been developed and shown to act more effectively than DCD and NP on AOA in acidic soils [132]. The isolation of acid-tolerant and acidophilic strains also offers opportunities for evaluating the efficacy of existing nitrification inhibitors on nitrification under acidic conditions, for understanding their mechanisms of action, as well as for designing and testing novel inhibitory compounds specific to the unique physiology of acidophilic nitrifiers while minimising harmful impacts on non-target organisms and the environment. Besides synthetic nitrification inhibitors, there is a prospect of employing biological nitrification inhibitors (BNIs) released in the rhizosphere of certain plants [131,133,134]. These plant-produced compounds could act as a natural control of nitrifier activities (reviewed in [135]), but it is unclear whether plants produce sufficient amounts of BNI for inhibiting nitrification when growing in acidic/alkaline soils or whether BNI can maintain their function in these soils.

### Wastewater treatment at low pH

Acidic nitrification has extensive implications in global wastewater treatment. Biological nitrogen removal from wastewater is based on nitrification and denitrification mediated by autotrophic nitrifiers in oxic tanks and heterotrophic denitrifiers in anoxic tanks, respectively. Compared to a generally low abundance and activity of AOA, AOB usually play a more critical role in converting ammonium to nitrite in these systems [136,137]. The dominant NOB genera in most wastewater treatment plants (WWTPs) are *Nitrospira* and *Nitrotoga* [137,138]. Compared with the conventional combination of nitrification and denitrification, the application of either partial nitritation coupled with the anaerobic oxidation of ammonium (anammox) (PN/A) or of the ‘nitrite shunt’ approach can reduce aeration costs by 60% and eliminate the need for organic carbon dosing to heterotrophic denitrifiers [139], thus being more sustainable nitrogen removal techniques. Anammox is a process carried out by Planctomycetes in the *Brocadiales* order that oxidize ammonium using nitrite as the electron acceptor to produce dinitrogen gas. In contrast, the nitrite shunt approach is the attempt to stop nitrification at nitrite, which is then reduced to dinitrogen gas. Both processes require the selective suppression of NOB while retaining the activities of AOB and of anammox or denitrifiers, respectively [140]. However, this this has been a long-standing technological challenge.

NOB are typically sensitive to acid stress (see above), which has inspired attempts to develop efficient nitrification at low pH using acid-tolerant AOB [37]. Acid-tolerant AOB can provide a stable source of nitrite for anammox bacteria, while suppressing the activity of NOB at low pH [37,141]. This coupling of acidic nitrification with anammox is a highly promising technology that has been demonstrated in both lab-scale [37,57,142,143] and pilot-scale reactors [144]. It can save significant aeration costs, eliminate the need for organic carbon, and allow for the separation and use of digested sludge in bioenergy recovery [141,145]. This technology has the potential to transform sewage treatment from an energy-negative to an energy-neutral service for wastewater treatment plants [139,146,147]. In acidic laboratory reactors treating urine, *Ca*. Nitrosacidococcus urinae was favored under acidic conditions, while NOB were likely absent [24]. Although aerobic acidic nitritation has the potential to brings tremendous energy sufficiency in wastewater treatment, N_2_O emissions during this process (likely caused by nitrite detoxification in the microbial consortia) cannot be ignored [123]. Currently, a better understanding of the mechanisms regulating N_2_O emission is needed for these highly promising systems that aim to operate with low energy consumption and environmental footprint.

## Conclusions and outlook

Through a combination of culture-based and culture-independent techniques, we now have an increasing understanding of the ecophysiology, adaptations, and distribution of nitrifying microorganisms in different ecosystems. As pH is intricately linked to substrate availability, bioenergetics and cellular defense against oxidative and nitrosative stress, it is crucial to deepen our understanding of mechanisms allowing nitrifiers to thrive or adapt to extreme pH. It is also critical to translate these ecological and physiological insights into mitigating nitrogen loss in agricultural land and improving energy efficiency in next-generation wastewater treatment processes.

## Summary

- Nitrification at acidic and alkaline pH has been understudied compared to nitrification at neutral pH conditions.
- Ammonia-oxidising bacteria and archaea, nitrite-oxidising bacteria, and comammox bacteria inhabit diverse acidic and alkaline environments.
- Physiological adaptations allow them to maintain pH homeostasis, acquire limiting energy and carbon sources, and detoxify toxic compounds at pH extremes.
- Nitrification at acidic pH contributes to nitrogen loss and nitrous oxide emissions in agricultural systems, but also has promising applications in acidic wastewater treatment.

## Open Access

Open access for this article was enabled by the participation of Monash University in an all-inclusive *Read & Publish* agreement with Portland Press and the Biochemical Society under a transformative agreement with CAUL.

## Abbreviations

AOA: ammonia-oxidising archaea
AOB: ammonia-oxidising bacteria
BNI: biological nitrification inhibitor
DCD: dicyandiamide
DMPP: dimethylpyrazole phosphate
HP/HB: hydroxypropionate/hydroxybutyrate
NOB: nitrite-oxidising bacteria
NP: nitrapyrin
WWTP: wastewater treatment plant
